# Association of Insomnia, Lipid Profile, and Lipid-Lowering Medications: A Narrative Review

**DOI:** 10.31083/RCM24978

**Published:** 2025-01-15

**Authors:** Fang Jia, Shun-Yi Shi, Si-Fan Fei, Min Zhou, Jian-Jun Li

**Affiliations:** ^1^Department of Cardiology, The Third Affiliated Hospital of Soochow University, 213003 Changzhou, Jiangsu, China; ^2^Cardio-Metabolic Center, Fu Wai Hospital, Chinese Academy of Medical Sciences, Peking Union Medical College, 10037 Beijing, China

**Keywords:** insomnia, lipid metabolism, coronary artery disease, statin, proprotein convertase subtilisin/kexin type 9 inhibitors (PCSK9i)

## Abstract

Sleep is a fundamental phenomenon that helps maintain normal physiological processes. Conversely, sleep disorders, usually presented as insomnia, are a common public health problem that can lead to multiple pathophysiological changes in humans, including lipid metabolic abnormality. Interestingly, several previous studies have examined the potential relation of insomnia to metabolic syndrome and hyperlipidemia and found that insomnia was associated with elevated plasma cholesterol and triglyceride concentrations. This review summarizes evidence regarding the linkage between insomnia and lipid abnormalities. Moreover, the underlying physiologic mechanisms linking insomnia to lipid abnormalities are systemically discussed. Finally, issues with lipid-lowering drugs and the risk of insomnia are also presented. This knowledge can improve our understanding of the pathophysiological features of insomnia, which may help to prevent and treat insomnia-induced dyslipidemia clinically.

## 1. Introduction

Sleep is a fundamental and intricate physiological activity essential for emotional and physical well-being that enables the human body to recover after previous activities and ensures body functioning during subsequent wakefulness [1]. Sleep is also a crucial indicator of general well-being and health outcomes associated with further health issues. Healthy sleep patterns and normative sleep durations are important to maintain good sleep quality and encourage full daytime alertness [2]. However, sleep is often overlooked. The time allotted to sleep and sleep quality have generally declined in the last few decades because people are increasingly curtailing their sleep or suffering from sleep deprivation as a reaction to working stress, social activities, depression, or other psychiatric disorders [3]. Further, abnormal sleep and deviations from optimal sleep duration can unhealthily affect physical and psychological health.

**Fig. 1.**
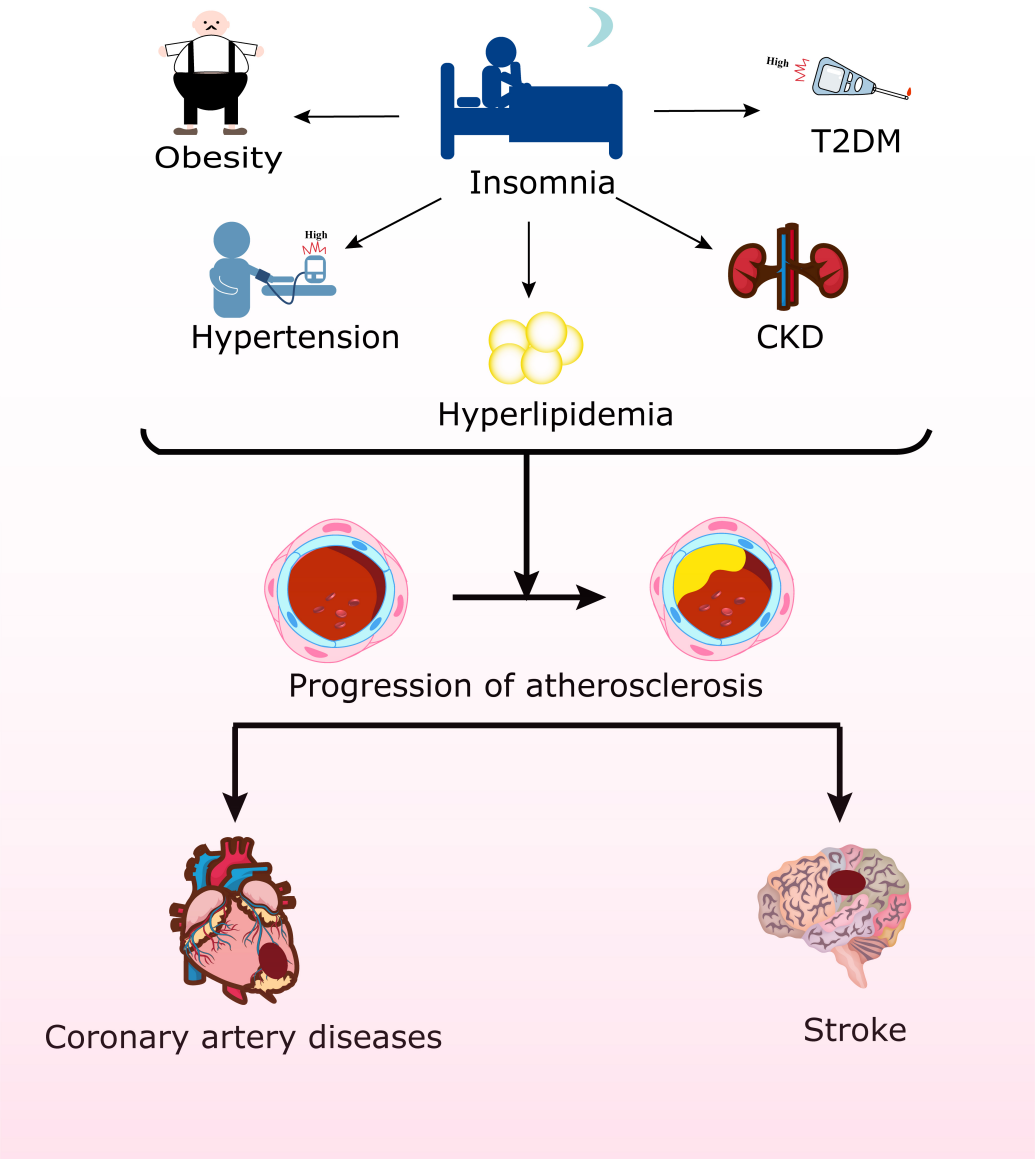
**Insomnia is correlated with obesity, hypertension, type 2 diabetes mellitus (T2DM), hyperlipidemia, and chronic kidney disease (CKD), further leading to coronary artery disease (CAD) and stroke**. The figure was created using Adobe Illustrator 2024.

Abnormal sleep patterns, such as insomnia, sleep fragmentation, sleep deprivation, and sleep-disordered breathing, have been acknowledged as a critical public issue leading to multiple organ diseases. Insomnia is also prevalent among the older population and indicates poor physical and psychological well-being [4]. Additionally, insomnia is associated with obesity [5], hypertension [6], type 2 diabetes [7], and chronic kidney disease [8], further leading to coronary artery diseases [9] and stroke [10] (Fig. 1). Moreover, people who have insomnia are at an increased risk of mortality through myocardial infarction and heart failure [11, 12, 13]. Recent data have demonstrated that sleep disorder-induced dyslipidemia may be important in the increased risk of cardiovascular diseases among insomniacs. Theoretically, the quality and duration of sleep influence lipid metabolism in several ways, including altering the endocrine regulation of hormones [14], impacting genes responsible for cholesterol transportation [15], causing a proinflammatory condition, and triggering depression or other psychiatric disorders. However, evidence of an association between insomnia and dyslipidemia remains inconsistent. This review summarizes the evidence regarding the linkage of insomnia with dyslipidemia and discusses the underlying mechanisms linking insomnia to lipid abnormalities. Issues relating to lipid-lowering drugs and the risk of insomnia are also discussed.

## 2. Methods

We performed a narrative review of the literature in the PubMed and Web of Science databases. These studies mainly focused on humans, while some animal studies were also included if the underlying mechanisms involved were considered potentially relevant and the findings closely related to those in the human studies. Cross-sectional, case-control, retrospective, and cohort human studies were included in our analysis. The following search terms were used: insomnia, sleep disorder, lipid, low-density lipoprotein-cholesterol (LDL-C), total cholesterol (TC), high-density lipoprotein-cholesterol (HDL-C), triglyceride (TG), hypothalamic–pituitary–adrenal (HPA) axis, cortisol, inflammation, depression, coronary artery disease, statin, proprotein convertase subtilisin/kexin type 9 inhibitors (PCSK9i), ezetimibe, nicotinic acid, fibrates, and omega-3 polyunsaturated fatty acids. A review of English-language publications was conducted. No time restrictions were imposed on eligible studies.

## 3. Definition and Epidemiology of Insomnia

Generally, insomnia is characterized by dissatisfaction with sleep on either a qualitative or quantitative level [16]. The Diagnostic and Statistical Manual of Mental Disorders, Fifth Edition (DSM-5), defines insomnia as an experience of insufficient or poor sleep quality associated with one or more of the following: (1) difficult sleep initiation, (2) difficult sleep maintenance, characterized by frequent awakenings or problems returning to sleep after awakenings, and (3) early-morning awakening with inability to return to sleep [17]. However, the third edition of the International Classification of Sleep Disorders (ICSD-3) is the authoritative guide for sleep disorder diagnoses. The ICSD-3 consolidates all insomnia diagnoses (i.e., “primary” and “comorbid”) under a single, chronic insomnia disorder in favor of a broad category for insomnia disorder [18]. Chronic insomnia diagnoses for ICSD-3 include (1) a report of sleep initiation or maintenance problems, (2) adequate opportunity and circumstances to sleep, and (3) daytime consequences [18]. In addition to the duration criterion (3 months), a frequency criterion (at least thrice weekly) was added to diagnose chronic insomnia.

As a significant public health problem, clinical care and health interventions should be provided for insomnia at both the individual and social levels [19]. Insomnia among the general public exhibits considerable variation in different research findings, ranging from 5% to 50% [19]. Such broad discrepancies may stem from the differences in defining insomnia, the assessment tools, and geographical locations. The worldwide prevalence rates of insomnia tend to cluster between 6% and 10% when using DSM or ICSD diagnostic criteria [19, 20]. A meta-analysis of the pooled prevalence of insomnia in China shows that the occurrence rate of insomnia within the Chinese population is 15.0% [21], which is lower than in several Western countries (e.g., 27.1% in the USA and 50.5% in Poland) [22, 23]. Many investigations have indicated a growing occurrence of insomnia with advancing age; however, the prevalence of insomnia patterns has mixed results [24], such as difficulty in maintaining sleep is commonly observed in adults who are in their middle age and beyond, whereas challenge with initiating sleep is more frequently encountered by younger adults [19]. Further, previous studies have shown that women are more prone to insomnia than men [25, 26]. A cross-sectional survey of sleep among Koreans showed that the prevalence of insomnia symptom subtypes, such as difficult sleep initiation, difficult sleep maintenance, and early-morning awakening, was also higher in women than in men [25]. Features of insomnia commonly consist of chronic duration [27, 28], impaired daytime performance, and deteriorating life quality. Data from one cohort study found that 41.6% of its participants experienced continuous insomnia throughout the entire duration of a 5-year follow-up [27]. Several studies support the connection between acute and chronic insomnia and the risk of cardiometabolic diseases, such as impaired glucose tolerance, diabetes, hypertension, and other cardiovascular issues [7, 11, 29]. However, further studies are needed to examine pathophysiological changes in cardiometabolic status during insomnia.

## 4. Sleep and Lipid Metabolism

**Fig. 2.**
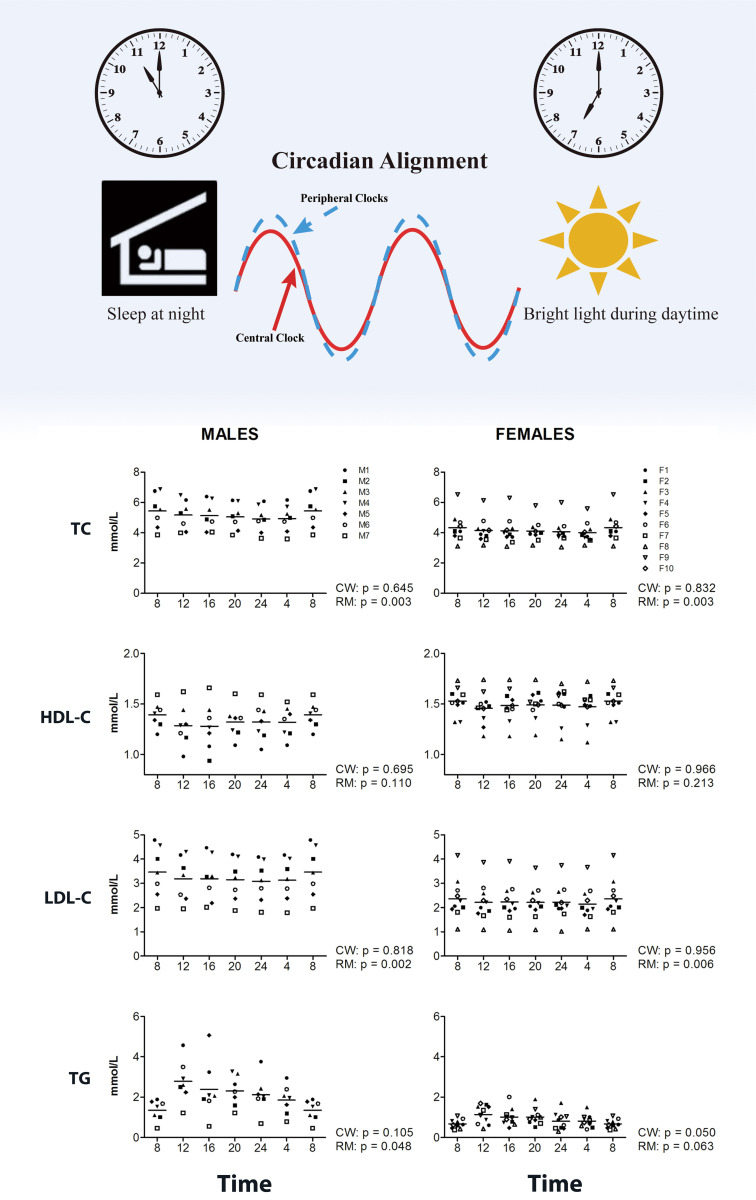
**Circadian rhythm and plasma lipids**. Levels of different lipids were measured throughout the day at four-hour intervals for male (left panels) and female (right panels) participants (adapted from van Kerkhof *et al*. [32]). Samples from 17 healthy volunteers, 10 women and 7 men, were analyzed in this study. Trends toward diurnal variations were observed for all lipid markers (TC, LDL-C, TG) except (HDL-C). Repeated measures ANOVA (RM ANOVA) showed significant effects of time for TC and LDL-C in both genders, for TG in males, and a trend was present in females. Abbreviations: TC, total cholesterol; LDL-C, low-density lipoprotein cholesterol; TG, triglyceride; HDL-C, high-density lipoprotein cholesterol; CW, CircWave Batch v5.0 Software analysis; RM ANOVA, repeated measures ANOVA. *p* < 0.05 was considered statistically significant. The figure was created using Adobe Illustrator 2024.

Metabolic processes are directed by the circadian system in daily 24-hour cycles. Lipid metabolism is extensively regulated by the circadian system [30, 31]. Several investigations have explored diurnal rhythms in cholesterol and other lipids in humans and found that they exhibited circadian variation over the day (Fig. 2, Ref. [32]). Although the acrophases and circadian amplitudes differed among different age groups [31], a pronounced circadian variation was observed in plasma TC and HDL-C concentrations among healthy Indians across various age groups [33]. Diurnal variations in LDL-C, TG, and TC in a study by van Kerkhof *et al*. [32] aligned with previous findings. Circadian rhythms were also confirmed in male participants by Cosinor analysis, which revealed significant diurnal rhythms of TC, which peaked in the early afternoon, TG peaking in the afternoon, and LDL-C peaking in the morning [34]. An important feature of circadian rhythms is that they need to be regularly entrained to maintain periodic rhythmicity (day–night circle); meanwhile, the intensity of the circadian response decreases over time without regular exposure [30]. An insomnia disorder can lead to circadian disruption, which in turn perpetuates insomnia [35]. One study on treatments found that re-timing the body clock successfully treated sleep onset insomnia, early morning awakening, and sleep maintenance insomnia [35].

Epidemiological data reveal that night work and deviation from mid-range sleep length are associated with changed lipid profiles [36, 37, 38]. Survey and biometric data revealed that long-haul truck drivers exhibited an increased atherosclerotic risk for their poor overall cholesterol profile [38]. Moreover, sleep variables such as sleep duration and workday sleep quality were powerful predictors of increased non-HDL-C, LDL-C, and TC in long-haul truck drivers, demonstrating that sleep homeostasis plays a role in regulating lipid metabolism. Circadian rhythm sleep–wake disorders (CRSWDs) were found to be correlated with a greater risk of dyslipidemia in railway workers in Southwest China [39]. Despite the separation of insomnia and CRSWDs in the diagnosis of sleep disorders implying independent pathophysiology, there is substantial proof of comorbidity and interplay between them [35].

Although the relationship between sleep and lipid metabolism could be affected by age, metabolic phenotype, meal times, behavioral, and other external factors [36, 38, 40, 41], genetic components might also regulate the relationship. Indeed, oscillations in the expression of genes that maintain lipid homeostasis are initially observed: (1) 3-hydroxy-3-methyl-glutaryl-coenzyme A reductase (HMGCR), a rate-limiting enzyme of cholesterol synthesis; (2) the low-density lipoprotein (LDL) receptor (LDLR), which mediates the transportation of cholesterol into hepatic cells; (3) cholesterol-7α-hydroxylase (Cyp7α1), performs cholesterol degradation [42]. Further, many other key enzymes involved in the intestinal absorption of lipids, hepatic lipid synthesis, and cholesterol transport are shown to be under circadian control, such as the farnesoid X receptor, the main regulator of bile acid synthesis, and ATP-binding cassette-binding proteins G5 (ABCG5), responsible for free cholesterol efflux towards the intestine [31]. In experimental mice, genes related to cholesterol metabolism are found to be differentially expressed between sleep and sleep-deprived groups [43], and impaired clock gene activity or circadian cycles results in reduced lipid homeostasis [31]. Second, sleep regulation and lipid metabolism potentially share the same genetic component, meaning genetic variants that influence blood lipid levels would also impact sleep duration [44]. Two genetic variants near the *TRIB1* gene, which participates in regulating cell cycle progression, are found to be independently involved in regulating lipid metabolism and sleep [44]. Interestingly, sleep and lipid metabolism may be mediated, at least partially, through the same genetic mechanisms; thus, the shared genetic background may explain the linkage between sleep and lipid metabolism.

## 5. Insomnia and Dyslipidemia

Epidemiologic evidence shows that poor or inadequate sleep leads to alterations in the metabolic processes of peripheral metabolism, which may lead to the occurrence of hyperlipidemia and even contribute to coronary heart disease (CHD) [45, 46]. In one cross-sectional study, people with insomnia symptoms in the last month had significantly elevated odds ratios of low HDL-C and high TG compared to healthy controls [47]. Several researchers have explored the correlation between insomnia and hyperlipidemia (Table 1, Ref. [47, 48, 49, 50, 51, 52, 53, 54, 55]).

**Table 1.** **Characteristics of the observational studies investigating lipid levels among individuals with insomnia symptoms**.

Author, y	Study design	Numbers	Location	Age (yrs)	Male (%)	Follow-up (y)	Insomnia testing method	Target variables	Variables categories	Outcomes assessed	OR; *p*
Syauqy, A. *et al*. 2019 [47]	Cross-sectional	26,016	Taiwan, China	≥35	64.9	-	Self-reported (questionnaire)	Sleep duration	<6 h/day (Ref: 6–8 h/day)	Low HDL-C (men)	1.239 (1.047–1.465)
										Low HDL-C (women)	1.061 (1.013–1.111)
										High TG	1.202 (1.065–1.358)
								Insomnia symptoms	Ref: no insomnia symptoms	Low HDL-C (men)	1.045 (1.005–1.085), 0.025
										Low HDL-C (women)	1.050 (1.011–1.090), 0.012
										High TG	1.066 (1.048–1.084), 0.000
Wang, Y. *et al*. 2017 [48]	Cross-sectional	8017	China	-	51.8	-	Self-administered questionnaire (Athens Insomnia Scale, AIS)	Insomnia symptoms	AIS Scores ≥6 (Ref: AIS Scores <6)	Low HDL-C	1.16 (1.01, 1.33), 0.04
										High TG	1.10 (0.94, 1.28), 0.25
Garbarino, S. and Magnavita, N. 2019 [49]	Prospective	234	Italy	36.0 ± 7.4	100	5	Self-administered questionnaire (Pittsburgh Sleep Quality Index, PSQI)	Insomnia symptoms	Ref: no insomnia symptoms	Low HDL-C	13.01 (2.41–70.12), 0.003
										High TG	108.93 (10.01–1178.34), 0.000
Tsiptsios, D. *et al*. 2022 [50]	Cross-sectional	957	Greece	49.62 ± 14.79	45.9%	-	Self-administered questionnaire	Sleep duration	<6 h/day (Ref: 6–8 h/day)	Dyslipidemia	2.18 (1.50–3.19), <0.001
								Delay in falling asleep	At least once a week (Ref: Less than once a week)		1.18 (0.85–1.63), 0.328
								Inability to maintain asleep			2.99 (2.05–4.36), <0.001
								Early morning awakenings			1.38 (1.00–1.89), 0.050
Zhang, Y. *et al*. 2022 [51]	Cross-sectional	1252	China	≥40	47.44	-	Self-administered questionnaire	Sleep duration	<6 h/day (Ref: 6–8 h/day)	Low HDL-C (men)	9.525 (5.488–16.530)
										Low HDL-C (women)	2.427 (1.550–3.801)
										High TG	11.779 (8.051–17.234)
								Insomnia symptoms	Ref: no insomnia symptoms	Low HDL-C (men)	2.054 (1.276–3.307), 0.003
										Low HDL-C (women)	1.222 (0.840–1.779), 0.295
										High TG	1.507 (1.132–2.007), 0.005
Yao, C. A. *et al*. 2023 [52]	Retrospective	1368	Taiwan, China	19–70	69.66	-	Self-administered questionnaire	Wake-up frequency	1/night	High non-HDL-C	1.15 (0.85, 1.56)
									(Ref: 0/night)	High TG	1.22 (0.86, 1.75)
										High TC	1.22 (0.90, 1.66)
									2/night	High non-HDL-C	1.50 (1.00, 2.24)
									(Ref: 0/night)	High TG	1.49 (0.94, 2.36)
										High TC	1.25 (0.83, 1.88)
									≥3/night	High non-HDL-C	1.78 (1.09, 2.89)
									(Ref: 0/night)	High TG	1.68 (0.99, 2.86)
										High TC	1.54 (0.93, 2.54)
Deng, H. B. *et al*. 2017 [53]	Prospective	162,121	China	20–80	47.4	18	Self-administered questionnaire	Sleep duration	<6 h/day (Ref: 6–8 h/day)	Low HDL-C	1.07 (1.03–1.11)
										High TG	1.09 (1.05–1.13)
Zhan, Y. *et al*. 2014 [54]	Cross-sectional	10,054	China	≥18	36.67	-	Self-administered questionnaire	Insomnia symptoms	Ref: no insomnia symptoms	High TC (men)	1.17 (0.86–1.58)
										High TC (women)	1.25 (1.05–1.50)
Silva-Costa, A. *et al*. 2020 [55]	Cross-sectional	13,722	Brazil	Women: 55.6	45.4	-	Self-reported (questionnaire)	Insomnia symptoms	Ref: no insomnia symptoms	Hypertriglyceridemia	Women: 1.17 (1.03; 1.34)
				Men: 55							Men: 1.17 (1.02; 1.35)

Abbreviations: TC, total cholesterol; TG, triglyceride; HDL-C, high-density lipoprotein-cholesterol; OR, odds ratio; y, year; yrs, years.

### 5.1 Duration of Insomnia

A 5-year prospective cohort study involving 242 police officers verified the causal link between insomnia symptoms and metabolic syndrome (MetS) [49]. A deterioration in all sleep indicators was noted during the follow-up: Sleep time declined from 6.7 ± 1.1 to 6.4 ± 1.2 hours (*p* < 0.001); police officers presenting with difficulty sleeping, sleep interruptions, or early awakenings increased from 12% to 21%. Sleep disorders were found to be significantly related to the incidence of MetS, while 4% of new cases were observed to have hypertriglyceridemia (HTG), and 5% had low HDL-C over the 5-year follow-up. An investigation into the correlation between insomnia symptoms and MetS components revealed a close linkage between insomnia and the incidence of dyslipidemia. In the longitudinal data presented by Morin *et al*. [27], insomnia syndrome was a persistent sleep disorder, with 41.6% of participants experiencing ongoing insomnia throughout the entire duration of the 5-year follow-up. Individuals with more severe insomnia were at an increased risk of continuous sleep disturbances. However, further studies are required to investigate the influence of the duration of long-term insomnia on abnormal blood lipid metabolism to determine the pathophysiological changes during chronic insomnia.

### 5.2 Insomnia Patterns

Regarding insomnia patterns, one cross-sectional study analyzed the correlation between insomnia subtypes and dyslipidemia among Greek adults in the primary care setting [50]. It was believed that the incidence of dyslipidemia was significantly correlated with insomnia, especially in relation to difficulties maintaining sleep and early morning awakenings. In a stratified analysis according to gender, higher odds for dyslipidemia were associated with difficulties in maintaining sleep and early morning awakenings among females but not males. In another study, the nightly wake-up frequency was found to have a significant synergistic effect with subjective insomnia and was significantly correlated with atherogenic lipid abnormality, including higher levels of TC and TG, as well as non-HDL-C [52]. Moreover, this work found that females were at a higher risk of dyslipidemia if they reported experiencing insomnia or increased wake-up frequencies compared to males. However, few studies exist on the relationship between different insomnia and plasma lipid phenotypes; meanwhile, more investigations on the characterization of insomnia patterns and their influence on lipid metabolism are also needed.

### 5.3 Short Sleep Duration

Insomnia and sleep duration are often entangled when evaluating the potential impacts of sleep disorders on lipid metabolism. Data from the National Health and Nutrition Examination Survey (NHANES) assumes that short sleep duration is non-linearly associated with low HDL-C/high TG levels [56]. In one community-based cohort study, the odds for meeting the increased TG criterion (TG ≥150 mg/dL or use of anti-hyperlipidemic medication) were elevated by 53% in short-duration sleepers (6–<7 h/night) compared to controls who slept 7 to 8 hours per night [57]. A cross-sectional study including 16,652 participants found that females who slept less than 5 hours a night were at a higher risk of hypercholesterolemia [58].

### 5.4 Severity of Insomnia Symptoms 

Few studies examined the linkage of insomnia symptom severity with plasma lipids. College students are more sensitive to stress and anxiety, and this results in elevated TG levels when they experience insomnia symptoms [59]. Indeed, insomnia was positively associated with TG after controlling for all covariates, and a greater degree of insomnia was related to higher TG levels [59]. Interestingly, maternal nightly sleep duration and sleep efficiency measured by actigraphy were significantly associated with TG levels in umbilical cord blood samples [60]. The degree of insomnia severity is commonly assessed using the Insomnia Severity Index (ISI), which is composed of seven items, including the severity of problems with sleep onset, sleep maintenance, and early morning awakening; satisfaction with current sleep pattern; interference with daily functioning; noticeability of impairment attributed to sleep problems; level of distress caused by sleep problems during the previous 2 weeks. The total scores range from 0 to 28, and a score of 10 was suggested in a prior study as the cut-off for insomnia [61]. Few studies directly assess the correlation between changes in ISI score and blood lipid levels. In one cohort of people with diabetes, mild and severe insomnia symptoms were not associated with LDL-C, HDL-C, and TG levels [62]. However, one study evaluated a cardiac rehabilitation (CR) program and revealed that greater ISI scores alleviating during the 12-week follow-up were associated with a greater decrease in TC levels in patients who completed the outpatient CR program [63]. Additionally, there was a trend toward a connection between alleviations in insomnia symptoms and reductions in LDL-C and TG levels, though it was not statistically significant [63].

### 5.5 Inconsistent Findings and Potential Reasons

Conflicting evidence remains concerning the interaction between insomnia and hyperlipidemia risk. One cross-sectional study incorporated participants who self-reported the frequency of difficulty falling asleep, prolonged nocturnal awakening, and undesired early morning awakening in the previous month [64]. No notable correlations were found between having any insomnia symptom at least five times over the preceding month and lipid abnormality (high LDL-C, low HDL-C, or high TG). In contrast, individuals with insomnia symptoms who were administering hypnotic medications had a higher propensity for elevated LDL-C levels. Zhan *et al*. [54] only observed a significant correlation between insomnia symptoms and TC levels in female participants, with no significant results for TG, LDL-C, and HDL-C levels noted alongside no remarkable association between insomnia symptoms and dyslipidemia among male participants. A meta-analysis, which incorporated 12 studies with 114,439 participants and follow-ups ranging from 200 days to 10 years, found that shorter sleep durations did not have a notable association with increased hyperlipidemia risk in adults [65]. These inconsistent results could be attributed to several distinct factors: First, the existing evidence was mostly cross-sectional, and many studies had small size and short-term follow-ups. Moreover, many were performed in Chinese populations. Hence, more large, randomized control studies are required to confirm the connection and clarify the clinical applicability of these findings. Second, insomnia was assessed by self-reported questionnaires or in-home polysomnography (PSG). Subsequently, discrepancies were noted in the results when measured using different methods, and some experts argued that PSG did not help assess insomnia because it did not correlate with the subjective perceptions of patients [66]. Third, some critical confounders, such as gender, sleeping pill use, and obstructive sleep apnea (OSA) comorbidity, should also be considered. The scarce experimental data suggest that hypnotic drugs have different effects on cholesterol and lipoproteins. Treatment with trazodone reduced TC levels after 6 weeks in an randomized controlled trial (RCT) [67], while in another RCT, venlafaxine and mirtazapine improved the LDL/high-density lipoprotein (HDL) ratio only in responders [68]. Zopiclone markedly decreased serum lipids in hyperlipidemic rats [69]. Notably, insomnia and OSA frequently co-occur [70]. OSA is believed to be an important confounder because it is closely correlated with pro-atherogenic dyslipidemia [71]. OSA patients present with elevated TC, LDL-C, TG, and reduced HDL-C levels [71]. Periodic limb movements are also a very common comorbidity of insomnia. The evidence supporting the link between periodic limb movements and dyslipidemia is limited. One cohort with 4138 OSA patients found that patients with periodic limb movements during sleep had higher TG and lower HDL-C levels, with no difference in TC and LDL-C levels [72].

## 6. Potential Mechanisms of Insomnia-Induced-Dyslipidemia

### 6.1 Hormonal Changes

Insomnia can be characterized as a state of cerebral hyperexcitability or hyperarousal, manifested as an increased whole-body metabolic rate during sleep alongside wakefulness and elevated cortisol secretion [73, 74]. Insomnia is correlated with upregulated HPA axis activity and cortisol levels [75]. Insufficient sleep quality and prolonged sleep latency have been shown to activate the HPA axis. Further, HPA hyperactivity might play a role in the correlation between sleep disorders and the risk of obesity, diabetes, and hyperlipidemia [76]. The level of cortisol secreted by insomniacs with a greater degree of sleep disturbance is higher than those with a lower degree of sleep disturbance [77]. The Passos GS group [78] observed a correlation between insomnia severity and morning cortisol level. Furthermore, the increase in cortisol levels could be more significant in insomniacs with objectively shorter sleep durations [75]. Children with insomnia symptoms and objective shorter sleep durations, reported by their parents, showed markedly elevated evening and morning cortisol levels [79]. Furthermore, chronic insomnia manifested as a state of a chronically hyperactive HPA axis, which led to a decreased HPA axis response to the corticotropin-releasing hormone (CRH), particularly in patients with objective sleep disturbance [80]. Vgontzas *et al*. [80] hypothesized that an attempt to rectify the hyperactive HPA axis via functional negative glucocorticoid feedback mechanisms could explain why insomniacs were fatigued and less vigorous in the daytime although emotionally and physiologically hyperaroused during the night [80]. Serum cortisol was connected with the prevalence of hyperlipidemia. In patients with type 2 diabetes mellitus (T2DM), elevated serum cortisol levels were coupled with increased LDL-C and TG and decreased HDL-C levels [81]. A significant positive correlation was found between cortisol and the TC/HDL ratio in undergraduate students experiencing examination stress [82]. During pregnancy, increased endogenous glucocorticoid speeds up fatty acid uptake, leading to upregulated fatty acid oxidation and TG synthesis in the liver, ultimately inducing liver lipid accumulation and HTG [83]. Moreover, a cross-sectional investigation using PSG-recorded sleep parameters showed that the number of arousals significantly predicted morning cortisol, TC, and LDL-C levels and the LDL/HDL ratio [84].

Additionally, shorter sleep durations or sleep restrictions are linked to higher ghrelin levels and decreased leptin levels [85, 86]. These endogenous hormones regulate appetite through the adenosine 5’-monophosphate (AMP)-activated protein kinase (AMPK)–hypothalamic fatty acid metabolism and peripheral adiposity through the sympathetic nervous system [87], thus altering peripheral lipid metabolism. It will be interesting to investigate the impacts of these hormone profile changes accompanied by insomnia on susceptibility to dyslipidemias. However, a prospective clinical trial including 13 women with primary chronic insomnia found that elevated midnight salivary cortisol concentrations in those insomniac women were not correlated with impaired glucose and lipids metabolisms [88]. Current evidence remains limited; thus, further studies are required to determine whether and how alterations in cortisol and other hormones in insomnia impact lipid metabolism.

### 6.2 Inflammatory Cytokines

Compared with individuals without insomnia, patients with insomnia symptoms had remarkedly increased odds ratios of high C-reactive protein (CRP) [47, 89]. Further, evidence suggests that the secretion pattern of proinflammatory cytokines such as serum tumor necrosis factor-α (TNF-α) and interleukin-6 (IL-6) are changed in individuals with insomnia [90, 91]. Research indicates that females might be especially susceptible to heightened inflammatory markers, primarily females with sleep disturbances [92, 93]. In a study by Nowakowski *et al*. [92], 295 peri- and postmenopausal women were recruited, and the results revealed that sleep efficiency was reversely associated with circulating levels of IL-6. Inflammation markers such as CRP, TNF-α, and IL-6 may represent a common physiological process linking shorter sleep duration and insomnia to mortality [94]. CRP, IL-6, and TNF were found to play a critical role in sleep quality changes in animals and humans, indicating that the inflammatory load impacts sleep quality [95, 96, 97]. Exercise training and diet regimen could cause both the improvement in sleep parameters and downregulation in CRP, IL-6, and TNF-α levels, as well as an increase in IL-10 levels, which suggests that anti-inflammatory effects are accompanied by improved sleep quality among subjects with chronic insomnia [98, 99].

Chronic inflammation and adipokines have been identified as the basis of insulin resistance and metabolic syndrome, which profoundly affect lipid metabolism [100, 101]. TNF-α can affect three key lipid metabolic processes, elevating plasma TG levels: adipose tissue lipoprotein lipase, hepatic fatty acid synthesis, and lipolysis [100]. Another experimental study found that TNF-α exposure in fasting mice provoked a decrease in HDL-C with a simultaneous increase in LDL-C [102]. It is well established that inflammation plays a critical role in the development and progression of coronary artery disease (CAD), and its function in metabolic diseases such as MetS and hyperlipidemia is also recognized [101]. Chronic insomnia and persistent systemic inflammation related to insomnia may be associated with lipid abnormalities and contribute to increased cardiovascular risk. However, data regarding the correlation between insomnia, systemic inflammation, and dyslipidemia remain rare. Thus, subsequent investigations are required to recognize the functional role of systemic inflammation in the association between insomnia and dyslipidemia.

### 6.3 Depression and Other Psychiatric Disorders

Ample research has shown that insomnia is not only a potential trigger but also a symptom of depression [103, 104]. Depression or other psychiatric disorders might act as mediators in the correlation between insomnia and lipid abnormality. An animal experiment found that depressive insomnia syndromes were inextricably linked to melatonin and 5-hydroxytryptamine (5-HT), and the pathophysiological process of depressive insomnia comorbidity involved impaired pineal melatonin secretion and hippocampal synaptic plasticity [105]. A melatonin deficit in young adult rats caused defective emotional responses and elevated circulating cholesterol levels [106]. Severe insomnia was correlated with a higher incidence of HTG in patients with current major depressive episodes (MDEs), but this connection was only found in women [107]. Females with severe insomnia had at least a 4-fold higher risk of HTG compared with those without severe insomnia. Moreover, women with severe insomnia and MDE commodities might be at a greater risk of HTG than women within the overall population. Indeed, treating insomnia has been demonstrated to improve not only sleep but also depression and metabolic function. One study that recruited 20 patients with asymptomatic seropositive human immunodefic-iency virus (HIV) and comorbid insomnia and depression showed that alleviations in insomnia were correlated with ameliorated depressive symptoms and favorable metabolomic changes [108]. An animal study has demonstrated the potential efficacy of melatonin agonists in sleep maintenance, depression, and metabolic profiles, with downregulated levels of TG, TC, and plasma glucose alongside upregulated HDL-C compared to controls [109]. One meta-analysis, including eight RCTs, suggested melatonin supplementation has significant effects on TG and TC levels and no significant effect on LDL-C and HDL-C levels [110]. Another meta-analysis, including 12 trials, showed that melatonin reduced LDL-C and TG levels, with no remarkable impact noted on the HDL-C level [111]. Since depressive insomnia comorbidity is common, additional research is required to elucidate the observed correlation between depressive insomnia comorbidity and hyperlipidemia.

**Fig. 3.**
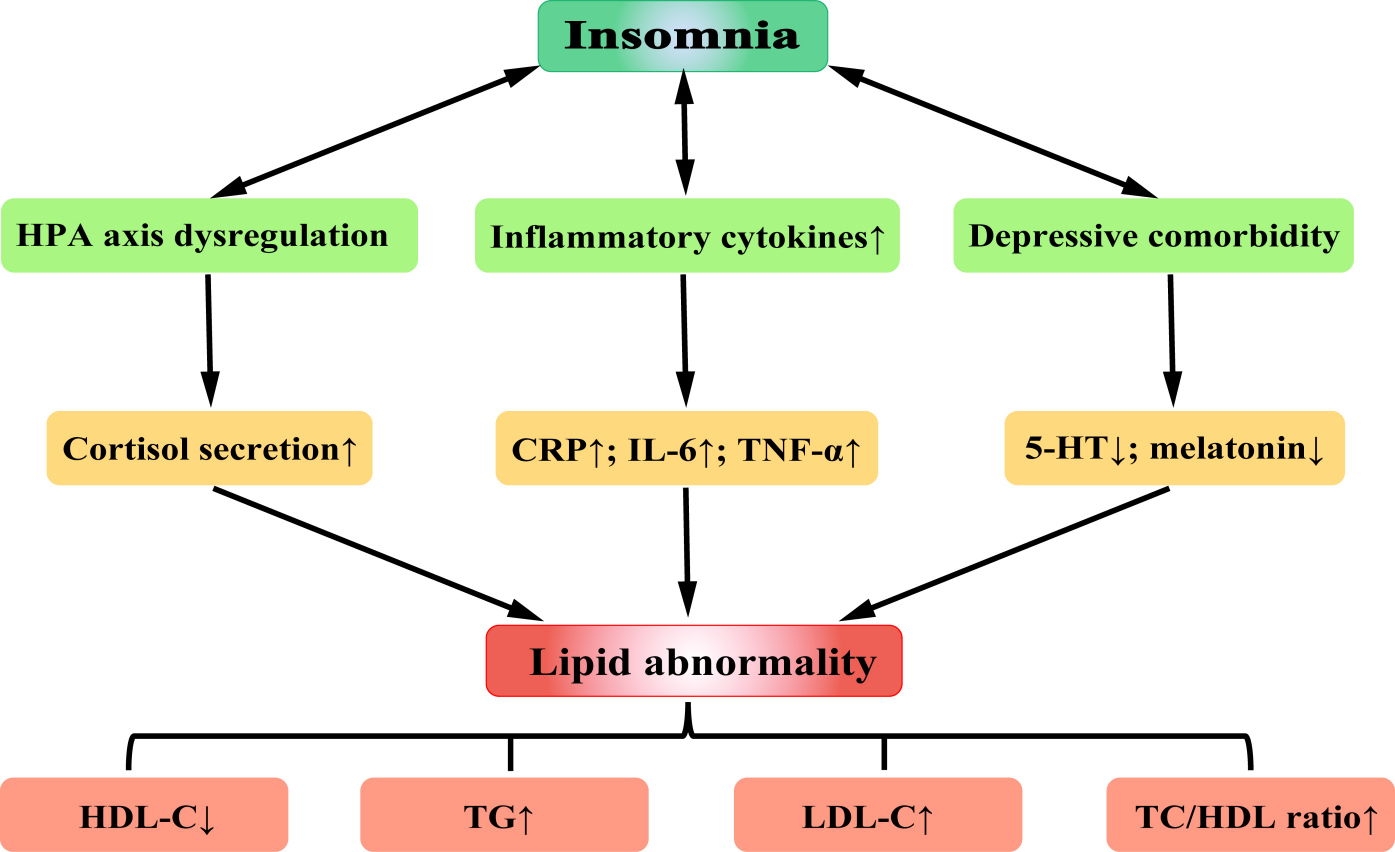
**Possible mechanisms for the correlation between insomnia and hyperlipidemia**. Abbreviations: HPA, hypothalamic-pituitary-adrenal; CRP, C-reactive protein; IL-6, interleukin-6; TNF-α, tumor necrosis factor-α; 5-HT, 5-hydroxytryptamine; HDL-C, high density lipoprotein-cholesterol; LDL-C, low density lipoprotein-cholesterol; TG, triglyceride; TC/HDL, total cholesterol/ high density lipoprotein. The figure was created using Adobe Illustrator 2024.

Complex interactions are involved between insomnia and HPA axis activity, inflammation, and depression. Insomnia potentially increases HPA axis activity, which could further intensify the existing concerns regarding insomnia and the associated daytime manifestations, setting in motion depression and anxiety [112]. The chronic process of insomnia and depression creates a proinflammatory condition, which could further change sleep patterns, behavior, and mood [113]. These compound factors, which are set in vicious circles, may further lead to lipid abnormality (Fig. 3).

## 7. Impact of Insomnia-Induced Dyslipidemia on CAD

Epidemiologic studies suggest a positive connection between insomnia and CAD [9, 13, 114].A population study enrolled 52,610 individuals and included 11.4 years of follow-up. After adjusting for age, sex, marital status, shift work, blood pressure, and other common risk factors, insomnia was found to be associated with a moderate increase in acute myocardial infarction (AMI) risk [13]. However, the potential influence of insomnia-induced dyslipidemia on CAD is not entirely clear. A cross-sectional study explored the association of metabolomics with CAD incidents in post-menopausal women and found that 58 lipid-related metabolites were elevated in women with poorer sleep quality following evaluation using the sleep questionnaire or Pittsburgh Sleep Quality Index (PSQI) [115]. Investigators created a sleep-related metabolite score (SMS) for each participant. For every standard deviation (SD) increase in SMS, the adjusted percentage difference in CAD biomarker levels was 4.1 for TC, –7.3 for HDL-C, 35.1 for TG, 22.7 for CRP, and 1.1 for fasting glucose, and every SD increase in SMS was correlated with 16% higher odds of developing CAD. Metabolomic profiling might offer crucial mechanistic insights into how poor sleep quality raises the risk of CAD through dysregulation of lipid metabolism [115]. As multiple risk factors contribute to CAD, it is very difficult to estimate the direct relevance of insomnia-induced dyslipidemia with CAD endpoints, given that evidence such as large cohort studies remains scarce. Insomnia is associated with various CAD risk factors, such as obesity, hypertension, diabetes, and hyperlipidemia, while the amelioration of insomnia is accompanied by an improvement in these risk factors [63]. Insomnia-induced dyslipidemia might be an important mechanism for the development of CAD based on the correlation mentioned above. Therefore, treating insomnia may reduce the occurrence and progression of CAD. Further studies are necessary to investigate whether interventions in alleviating insomnia could ameliorate dyslipidemia and modify metabolic processes that reduce CAD risk.

## 8. Lipid-Lowering Drugs and Insomnia Risk 

**Fig. 4.**
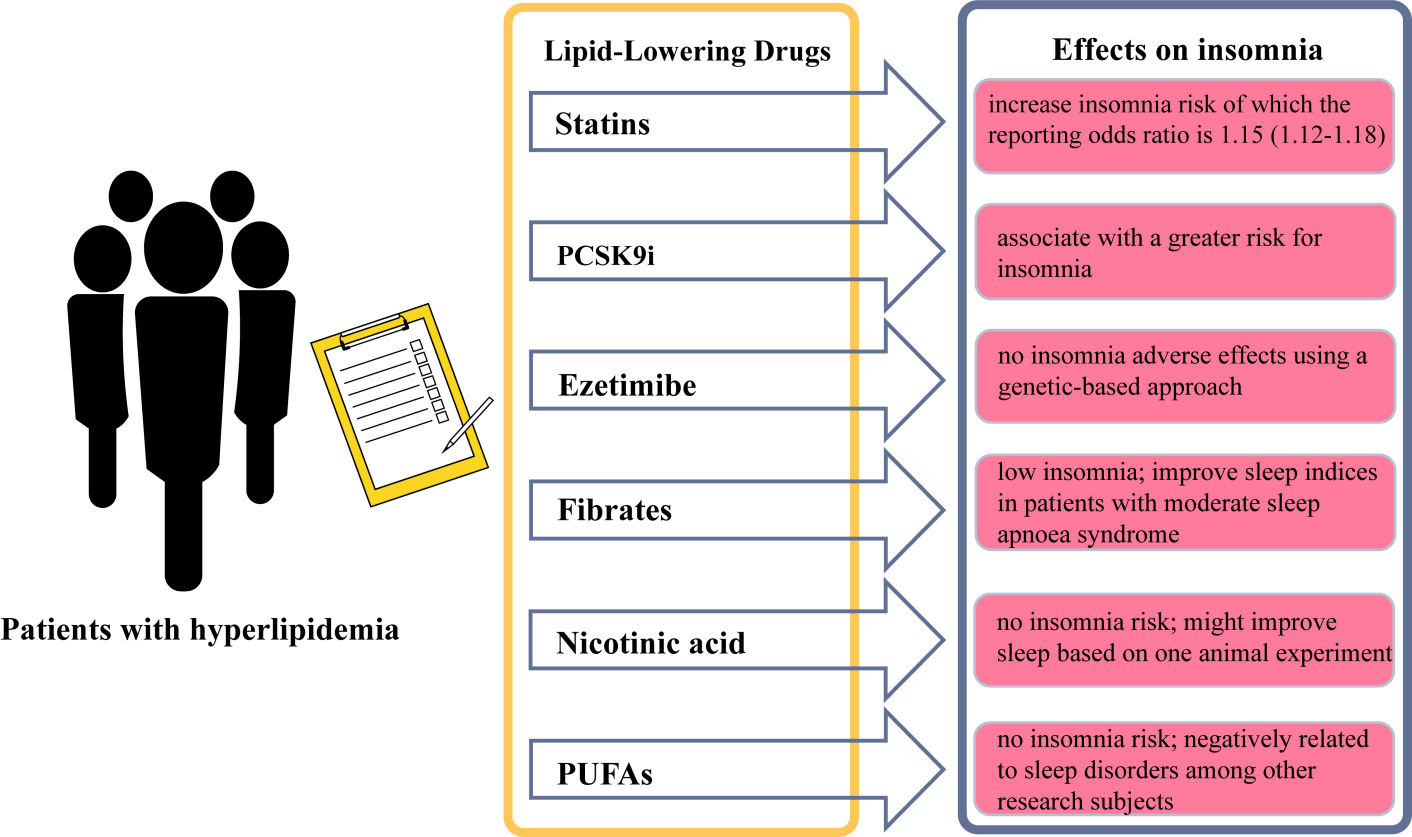
**Different lipid-lowering drugs and the risk of insomnia**. Abbreviations: PCSK9i, proprotein convertase subtilisin/kexin type 9 inhibitors; PUFAs, polyunsaturated fatty acids. The figure was created using Adobe Illustrator 2024.

Lipid-lowering medications are widely used and form a cornerstone of the cardiovascular risk modification strategy. Statins are predominantly used as the first-line lipid-lowering medication; however, many patients require other or additional lipid-lowering strategies to intensify the control of their lipid levels [116]. The occurrence of neurocognitive adverse events associated with lipid-lowering drugs remains a topic of contention [117]. Indeed, particular interest has been drawn to the potential impact of lipid-lowering medications on sleep quality (Fig. 4). With the emergence of novel lipid-lowering drugs, a better understanding of the correlation between drug administration and insomnia might help determine the optimal choice of appropriate preventive medications to improve the clinical outcomes in hyperlipidemia patients.

### 8.1 Statins

An investigation by Takada *et al*. [118] indicated that statin use was linked to a higher risk of sleep disruptions, including insomnia. Researchers have analyzed data using the US Food and Drug Administration Adverse Event Reporting System (FAERS) and a prescription database constructed by JMIRI (Japan Medical Information Research Institute, Inc. Japan). Moreover, different statins might have a varying extent of sleep disturbance risk. In the analysis of individual statins, simvastatin, rosuvastatin, and lovastatin showed significant signals in initiating and maintaining sleep in patients with disorders; meanwhile, atorvastatin and pitavastatin did not provide a significant signal in patients with sleep disorders. In the JMIRI prescription database, hypnotic drug use was found to exhibit a notable correlation with the entire category of statins but not with individual statins [118]. A cross-sectional cohort study conducted by Alsehli and colleagues [119] found that statin users were at a higher risk of insomnia compared to controls, suggesting that statin therapy might increase insomnia risk in the European population. The plausible pharmacological mechanism for statins in insomnia is unknown. Although lipophilic statins are proposed as being related to a higher rate of central nervous system disorders compared to hydrophilic statins [120], the investigation by Takada *et al*. [118] does not support the hypothesis that patients administering hydrophilic statins are less likely to suffer from insomnia. The HMGCR, the pharmacological target of statin, is highly conserved in humans and Drosophila. Using Drosophila, Alsehli *et al*. [121] discovered that suppressing the HMGCR in specific *pars intercerebralis* neurons within the Drosophila brain was sufficient to disrupt normal sleep patterns. The particular function of the HMGCR in managing sleep behavior is interesting and requires further studies to explore the connected molecular basis. Furthermore, two previously identified off-targets of statins, the peroxisome proliferator-activated receptor alpha (PPARα) (activated by statins) [122] and complex III in the mitochondrial respiratory chain (inhibited by statins) [123], are speculated to be related to the insomnia-promoting effect of statin treatment.

Individual statins with distinct tissue distribution characteristics may affect sleep behavior differently [119]. Nevertheless, no substantial evidence has revealed a particular statin that is more prone to be correlated with insomnia over others; thus, switching to another statin can alleviate symptoms in some cases but not in others [118, 124]. Furthermore, the predominant portion of statin users in the study by Alsehli *et al*. [119] had prescriptions for simvastatin, which limits the conclusions to be drawn to the statin pharmacology. Broncel *et al*. [125] performed a meta-analysis that included five eligible studies and 231 male participants to analyze the impact of statin use on sleep parameters using PSG. Their findings showed that statin use had no adverse effect on sleep duration and efficiency. Furthermore, statin use could alternatively promote some positive effects, such as reducing wake time and the number of awakenings. Simply, statin use remains inconclusive since previous results are inconsistent with the correlation between statin use and insomnia. Further prospective, randomized controlled trials, which consider statin type, patient age, gender, and race, are required to testify to the causality between insomnia and statin use.

### 8.2 PCSK9i

PCSK9i are currently emerging as an outstanding therapeutic option for reducing cardiovascular disease risk. However, there remains concern about PCSK9i therapy regarding the possible negative effects on neuropsychiatric function since *in vitro* and *in vivo* studies demonstrate that proprotein convertase subtilisin/kexin type 9 (PCSK9) is involved in a range of neural processes [126, 127]. In an analysis by di Mauro *et al*. [128], which was based on the European pharmacovigilance database, the most commonly reported adverse drug reactions (ADRs) for alirocumab and evolocumab were headache, insomnia, and depression; meanwhile, no difference was observed between alirocumab and evolocumab use and the probability of neuropsychiatric ADRs. It should be acknowledged that females are more prone to encounter ADRs independently by drug classes. The prevalence of insomnia among the occurrence of ‘psychiatric disorders’ for alirocumab was 11.84%, while it was 8.76% for evolocumab [128]. PCSK9 genetic variants that could mimic PCSK9i effects were used and found to be linked with a greater risk of insomnia. Further investigations are necessary to estimate the impact of PCSK9Is on insomnia and other neurocognitive functions.

### 8.3 Ezetimibe

Using a genetic-based approach, no significant neuropsychiatric adverse effects, including insomnia, were associated with the genetic risk score of *NPC1L1* (drug target gene of ezetimibe); moreover, a sensitivity analysis showed no evidence of heterogeneity or directional pleiotropy in all the analyses [129]. This study provides data based on genetic surrogates of ezetimibe targets, revealing that no insomnia effect is associated with ezetimibe treatment. However, more clinical analyses are needed in larger populations to confirm the impact of ezetimibe administration on sleep efficiency.

### 8.4 Fibrates and Nicotinic Acid

Few studies have evaluated the safety profile of fibrates on insomnia. One study, which applied a follow-up of over 5.3 ± 4.7 years (range: 1 to 16 years), assessed the real-life efficacy and adverse effects of fibrate treatment for HTG [130]. They concluded that long-term fibrate therapy was safe because a total of 8 (4.3%) patients had adverse effects, including three with liver enzyme elevations, two with myalgia, one with insomnia, one with malaise, and one with a skin condition. A proof-of-concept study suggested that fenofibrate might improve sleep parameters in overweight hypertriglyceridemic patients with moderate sleep apnea syndrome [131]. Nicotinic acid, another anti-hyperlipidemic drug used to improve plasma lipid profiles, especially HTG, is a member of the B3 vitamin group. Prolonged sleep deprivation may impair some aspects of nicotinic acid metabolism, and nicotinic acid depletion could cause sleep deprivation-induced neuronal damage [132]. The regulation of nicotinic acid on lipid metabolism interacts with multiple sleep modulation pathways, and one animal experiment found that intraperitoneal and oral gavage administration of nicotinic acid elicits robust increases in non-rapid-eye movement sleep (NREMS) in mice [133]. Therefore, taking nicotinic acid supplements may serve as a sleep aid based on these findings. However, more observational evidence is needed to characterize the impacts of fibrates and nicotinic acid on insomnia in humans.

### 8.5 Omega-3 Polyunsaturated Fatty Acids

Omega-3 polyunsaturated fatty acids confer cardiovascular benefits through TG reduction, anti-inflammatory and anti-arrhythmic effects, vasodilation, endothelial function improvements, etc. [134]. A factorial clinical trial was conducted using 168 female participants of reproductive age with pre-diabetes and hypovitaminosis D, and a notable improvement was observed in the sleep quality score in the omega-3 and omega-3 and vitamin D co-supplementation groups [135]. One systematic review and meta-analysis concluded that administering omega-3 long-chain polyunsaturated fatty acid (LC-PUFA) may decrease the total sleep disturbance score for children with clinical levels of sleep disorders; however, it does not influence the sleep results for children and adults without sleep problems [136]. One cross-sectional study suggested that the intake of omega-3 fatty acids had an inverse correlation with sleep disorders in men; meanwhile, dietary intake of omega-3 fatty acids was negatively related to very short, short, and long sleep durations [137]. Currently, most studies focus on the correlation between dietary intake or supplementation of omega-3 polyunsaturated fatty acids and sleep parameters, while the effects of high dose omega-3 polyunsaturated fatty acids, especially icosapent ethyl, on sleep quality in patients with hypertriglyceridemia and CAD still need additional investigation. Subsequent research should consider the participants’ health status, gender, the eicosapentaenoic acid (EPA)/docosahexaenoic acid (DHA) content, and other potential confounders [136].

## 9. Conclusions 

Sleep is crucial for the normal function of metabolic and hormonal processes. Sleep duration and quality are correlated with lipid homeostasis. Epidemiological studies demonstrate that insomnia is connected with obesity, hypertension, metabolic syndrome, CAD, and stroke. Several studies have evaluated the correlation between insomnia symptoms and dyslipidemia and show that there is a strong relationship between insomnia and hyperlipidemia risk. Abnormal lipid metabolism may be an important pathogenic pathway linking insomnia with the risk of CAD. There is a complex interaction among insomnia, HPA axis activity, inflammation, and depression, which forms a vicious cycle and exacerbates dyslipidemia. The relationships between insomnia and dyslipidemia/CAD require further investigation, including examining the potential impact of efforts to alleviate insomnia on lipid abnormality and whether improving lipid metabolism and other CAD risk factors may bolster sleep quality. A comprehensive understanding of the connection between insomnia and lipid metabolism can optimize treatment strategies, thereby improving the quality of life and prognosis for insomnia patients.
